# Multi-scale systems genomics analysis predicts pathways, cell types, and drug targets involved in normative variation in peri-adolescent human cognition

**DOI:** 10.1093/cercor/bhad142

**Published:** 2023-04-27

**Authors:** Shraddha Pai, Shirley Hui, Philipp Weber, Soumil Narayan, Owen Whitley, Peipei Li, Viviane Labrie, Jan Baumbach, Anne L Wheeler, Gary D Bader

**Affiliations:** The Donnelly Centre, University of Toronto, Toronto, Canada; Adaptive Oncology, Ontario Institute for Cancer Research, Toronto, Canada; Department of Medical Biophysics, University of Toronto, Toronto, Canada; The Donnelly Centre, University of Toronto, Toronto, Canada; Department of Mathematics and Computer Science, University of Southern Denmark, Odense, Denmark; The Donnelly Centre, University of Toronto, Toronto, Canada; The Donnelly Centre, University of Toronto, Toronto, Canada; Department of Molecular Genetics, University of Toronto, Toronto, Canada; Center for Neurodegenerative Science, Van Andel Research Institute, Grand Rapids, MI, United States; Division of Psychiatry and Behavioral Medicine, College of Human Medicine, Michigan State University, Grand Rapids, MI, United States; Center for Neurodegenerative Science, Van Andel Research Institute, Grand Rapids, MI, United States; Division of Psychiatry and Behavioral Medicine, College of Human Medicine, Michigan State University, Grand Rapids, MI, United States; Department of Mathematics and Computer Science, University of Southern Denmark, Odense, Denmark; TUM School of Life Sciences Weihenstephan, Technical University of Munich, Munich, Germany; Neurosciences and Mental Health, The Hospital for Sick Children, Toronto, Canada; Department of Physiology, University of Toronto, Toronto, Canada; The Donnelly Centre, University of Toronto, Toronto, Canada; Department of Molecular Genetics, University of Toronto, Toronto, Canada; Department of Computer Science, University of Toronto, Toronto, Canada; The Lunenfeld-Tanenbaum Research Institute, Mount Sinai Hospital, Toronto, Canada

**Keywords:** genetics of cognition, pathway analysis, adolescence, noncoding variation, neuroimaging

## Abstract

An open challenge in human genetics is to better understand the systems-level impact of genotype variation on developmental cognition. To characterize the genetic underpinnings of peri-adolescent cognition, we performed genotype–phenotype and systems analysis for binarized accuracy in nine cognitive tasks from the Philadelphia Neurodevelopmental Cohort (~2,200 individuals of European continental ancestry aged 8–21 years). We report a region of genome-wide significance within the 3′ end of the *Fibulin-1* gene (*P* = 4.6 × 10^−8^), associated with accuracy in nonverbal reasoning, a heritable form of complex reasoning ability. Diffusion tensor imaging data from a subset of these participants identified a significant association of white matter fractional anisotropy with *FBLN1* genotypes (*P* < 0.025); poor performers show an increase in the C and A allele for rs77601382 and rs5765534, respectively, which is associated with increased fractional anisotropy. Integration of published human brain-specific ’omic maps, including single-cell transcriptomes of the developing human brain, shows that *FBLN1* demonstrates greatest expression in the fetal brain, as a marker of intermediate progenitor cells, demonstrates negligible expression in the adolescent and adult human brain, and demonstrates increased expression in the brain in schizophrenia. Collectively these findings warrant further study of this gene and genetic locus in cognition, neurodevelopment, and disease. Separately, genotype-pathway analysis identified an enrichment of variants associated with working memory accuracy in pathways related to development and to autonomic nervous system dysfunction. Top-ranking pathway genes include those genetically associated with diseases with working memory deficits, such as schizophrenia and Parkinson’s disease. This work advances the “molecules-to-behavior” view of cognition and provides a framework for using systems-level organization of data for other biomedical domains.

## Introduction

The growth in genomics and functional annotation resources over the past decade provides an opportunity to build models of how changing genotype affects multiple levels of system organization underlying a phenotype, from genes and molecules through to pathway, cell, cell circuit, anatomy, and physiology system levels (systems genomics analysis). This opportunity complements a conceptual shift to systems-level thinking in many biomedical fields. For example, a major drive-in psychiatry is the reconceptualization of mental illnesses as brain disorders treatable by neurobiological system-grounded therapies, such as working memory deficits in schizophrenia (Lett et al. 2014). As a shared guide for the field, the US National Institute of Mental Health has developed a “genes-to-behavior” framework that deconstructs human behavior into neurobehavioral domains, such as cognition and social processing (Insel et al. 2010). Each of these constructs has subconstructs and these are linked to a variety of systems level concepts. While the genetic architecture of overall cognitive ability (i.e. intelligence) has been studied by large-scale genome-wide association analysis (GWAS) (Okbay et al. 2016; Sniekers et al. 2017; Hill et al. 2019), little is known about the molecular basis of more detailed neurocognitive phenotypes.

In this work, we identify genetic variants associated with normative variation in peri-adolescent cognition, as measured by the Philadelphia Neurodevelopmental Cohort (PNC) (Calkins et al. 2014). We selected this study for our systems genomics analysis as the phenotypes measured activate specific neuroanatomical networks and are impaired in disorders of neurodevelopmental origin with significant heritability. For example, tasks requiring use of working memory, a type of short-term memory that recruits a cortical–subcortical network including the dorsolateral prefrontal cortex, shows a genetic component in twins, and is impaired in schizophrenia (Braver et al. 1997; Blokland et al. 2008; Minzenberg et al. 2009). With a standardized neurocognitive test battery and genotyping on over 8,000 community youths aged 8–21 years, the PNC is the largest publicly available dataset for genotype-phenotype analysis of peri-adolescent cognition (Gur et al. 2014; Satterthwaite et al. 2014; Calkins et al. 2015). Moreover, the PNC captures the age range through which some cognitive abilities, such as working memory, mature to stable adult levels (Luna et al. 2004; Simmonds et al. 2017). Phenotypes in the test battery have neurobehavioral validity (Gur et al. 2010), single nucleotide polymorphisms (SNP)-based heritability (Robinson et al. 2015), and disease relevance (Gur et al. 2010; Germine et al. 2016). Multiple cognitive test scores in the PNC demonstrate significant SNP-based heritability (Robinson et al. 2015), and reduced test scores are correlated with increased genetic risk of psychiatric disease (Germine et al. 2016). Thus, we hypothesize that this data will identify genetic variants linked to one or more system level scales of phenotype-related organization.

Despite the relatively small size of the PNC dataset from the perspective of GWAS studies, we reasoned that the availability of a well-validated cognitive test battery along with genetic and multi-modal brain imaging data provides a unique opportunity to study the molecular and systems basis of cognitive tasks impaired in neurodevelopmental disorders, during peri-adolescence. We also wished to evaluate how a systems genomics approach can increase statistical and interpretive power compared to standard SNP and gene-based analysis approaches, both of which are performed here to enable us to compare these approaches. To our knowledge, there have been no reports of genotype-phenotype analyses on the PNC dataset. Using diverse functional genomics resources, we link variants to genes, pathways, brain cell types, brain systems, predicted drug targets, and diseases, providing a systems-level view of the genetics of the neurodevelopmental phenotypes under study.

## Materials and methods

Cognitive assessment was performed using the Penn Computerized Neurocognitive Battery (CNB), which was customized and shortened for a pediatric population (Gur et al. 2014). Performance is measured by a session of trials containing items with varying levels of difficulty, which allows the test to capture nuances in speed and accuracy measures. Tests were also developed through evaluation by psychological investigators to ensure tasks could measure the phenotype of interest (i.e. had “construct validity”) and reliability between test-takers and through retakes (Gur et al. 2010).

### Genetic imputation

The samples (*n* = 8,719) were all genotyped using Illumina or Affymetrix SNP-array platforms by the Center for Applied Genomics at The Children’s Hospital of Philadelphia (Glessner et al. 2010). The workflow for genomic imputation is shown in Supplementary Fig. 1. Genotypes for the four most frequent microarray genotyping platforms were downloaded from dbGaP (phs000607.v1). We performed genetic imputation for the Illumina Human610-Quad BeadChip, the Illumina HumanHap550 Genotyping BeadChip v1.1, Illumina HumanHap550 Genotyping BeadChip v3, and the Affymetrix AxiomExpress platform (Supplementary Table 1, total of 6,502 samples before imputation), using the protocol recommended by the EMERGE consortium (Verma et al. 2014). Imputation was performed as follows:

#### Step 1: Platform-specific plink quality control

Quality control was first performed for each microarray platform separately. SNPs were limited to those on chr1-22. SNPs in linkage disequilibrium (LD) were excluded (—indep-pairwise 50 5 0.2), and alleles were recoded from numeric to letter coding. Samples were excluded if they demonstrated heterozygosity >3 standard deviations (SD) from the mean, or if they were missing > = 5% genotypes. Where samples had pairwise Identity by Descent > 0.185, one of the pair was excluded. Variants with minor allele frequency (MAF) < 0.05 were excluded, as were those failing Hardy-Weinberg equilibrium with *P* < 1e – 6 and those missing in >= 5% samples.

#### Step 2: Convert coordinates to hg19

LiftOver (Hinrichs et al. 2006) was used to convert SNPs from human genome assembly version hg18 to hg19; Hap550K v1 data was in hg17 and was converted from this build to hg19.

#### Step 3: Strand-match check and prephasing

ShapeIt v2.r790(Delaneau et al. 2013) was used to confirm that the allelic strand in the input data matched that in the reference panel; where it did not, allele strands were flipped (shapeit “–check” flag). ShapeIt was used to prephase the variants using the genetic_b37 reference panel (downloaded from the Shapeit website, http://www.shapeit.fr/files/genetic_map_b37.tar.gz).

#### Step 4: Imputation

Genotypes were imputed using Impute2 v2.3.2 (Howie et al. 2009) and a reference panel from the 1,000 Genomes (phase 1, prephased with Shapeit2, no singletons, 16 2014 June release, downloaded from https://mathgen.stats.ox.ac.uk/impute/data_download_1000G_phase1_integrated_SHAPEIT2_16-06-14.html) was used for imputation, using the parameter settings “–use_prephased_g –Ne 20000 –seed 367946”. Average concordance for all chromosomes was ~ 95%, indicating successful imputation (Supplementary Fig. 2). Imputed genotypes were merged across all platforms using software from the Ritchie lab (Verma et al. 2014) (impute2-group-join.py, from https://ritchielab.org/software/imputation-download) and converted to plink format. Following previous PNC genotype analysis (Robinson et al. 2015), only SNPs with info score >0.6 were retained, and deletions/insertions were excluded (plink “-snps-only just-acgt” flags). As preliminary quality control, when merging across chromosomes, samples with missingness exceeding 99% were excluded, as were SNPs with MAF <1% and with missingness exceeding 99%. This step resulted in 10,845,339 SNPs and 6,327 individuals.

#### Step 5: Post-imputation quality control

The HapMap3 panel was used to assign genetic ancestry for samples, using steps from (Anderson et al. 2010) (Supplementary Fig. 3). Individuals within five SD of the centroid of the HapMap3 CEU (Utah residents with Northern or Western European ancestry) or TSI (Tuscans in Italy) clusters were assigned to belong to the respective groups and were classified as being of European descent; 3,441 individuals pass this filter. Individuals with >5% missing data were excluded, as was one of each pair of individuals with Identity by State (IBS) >0.185 (47 individuals); 3,394 individuals passed this filter. Variants that were symmetric or in regions of high LD (Supplementary Table 2) were excluded (9,631,316 SNPs passed). Variants with >5% missingness were excluded (1,569,407 SNPs excluded). Finally, SNPs with MAF < 0.01 (3,168,339 SNPs) and failing Hardy-Weinberg equilibrium with *P*-value <1e – 6 (373 SNPs) were excluded, resulting in 4,893,197 SNPs. As only high-quality SNPs were retained after imputation, post-processing steps were performed only once. In sum, the imputation process resulted in 3,394 individuals and 4,893,197 SNPs available for downstream analysis.

### Phenotype processing

Phenotype data were downloaded from dbGaP for 8,719 individuals. In total, 637 individuals with severe medical conditions (Medical rating = 4) were excluded to avoid confounding the symptoms of their conditions with performance on the cognitive tests (Calkins et al. 2014, 2015; Robinson et al. 2015). Linear regression was used to regress out the effect of age at test time (variable name: “age at cnb”) and sex from sample-level phenotype scores, and the residualized phenotype was used for downstream analysis.

The nine phenotypes selected for systems genomics analysis are measures of overall performance accuracy in the Penn Computerized Neurocognitive Test Battery (CNB; Supplementary Table 3) and represent major cognitive domains. Tasks mapped to domains in the following manner: verbal reasoning, nonverbal reasoning, and spatial reasoning measured complex cognition; attention allocation and working memory measured executive function; recall tests for faces, words and objects measured declarative memory, and emotion identification measured social processing. Following regression, none of the phenotypes were significantly correlated with age after Bonferroni correction, indicating that the age effect had been reduced (Supplementary Table 4). Following guidelines from previous analyses on these data (Germine et al. 2016), individuals with scores more than four SD from the mean for a particular test, representing outliers, were excluded from the analysis of the corresponding phenotype. For a given phenotype, only samples with a code indicating a valid test score (codes “V” or “V2”) were included; e.g. for pfmt_tp (Penn Face Memory Test), only samples with pfmt_valid = “V” or “V2” were retained; the rest had scores set to NA. Finally, each phenotype was dichotomized so that samples in the bottom 33rd percentile were relabeled as “poor” performers and those in the top 33rd were set to be “good” performers; for a given phenotype, this process resulted in ~1,000 samples in each group (Supplementary Table 3). Where an individual had good or poor performance in multiple phenotypes, they were included in the corresponding group for each of those phenotypes.

### Genetic association analysis

For each of nine CNB phenotypes, marginal SNP-level association was calculated using a mixed-effects linear model (MLMA), using the leave-one-chromosome-out (LOCO) method of estimating polygenic contribution [GCTA v1.97.7beta software (Yang et al. 2011)]. In this strategy, a mixed-effect model is fit for each SNP:

*y* = *a* + b*x* + *g- + e.*

where *y* is the binarized label (good/poor performer on a particular task), *x* measures the effect of genotype (indicator variable coded as 0, 1, or 2), *g-* represents the polygenic contribution of all the SNPs in the genome (here, the ~4.89 M imputed SNPs), and *e* represents a vector of residual effects. In the LOCO variation, *g-* is calculated using a chromosome-specific genetic relatedness matrix, one that excludes the chromosome on which the candidate SNP is located (Yang et al. 2011). SNPs and associated genes were annotated as described in Supplementary Notes 1–4.

### Hi-C data processing

We downloaded publicly available higher-order chromatin interaction (Hi-C) data from human prefrontal cortex tissue (Schmidt et al. 2014; Schmitt et al. 2016) [Illumina HiSeq 2000 paired-end raw sequence reads; *n* = 1 sample; 746 Million reads; accession: GSM2322542 (https://www.ncbi.nlm. nih.gov/geo/query/acc.cgi?acc=GSM2322542)]. We used Trim Galore (v0.4.3) for adapter trimming (Martin 2011), Hi-C User Pipeline (HiCUP) (Wingett et al. 2015) (v0.5.9) for mapping and performing quality control, and GOTHIC (Mifsud et al. 2017) for identifying significant interactions (Bonferroni *P* < 0.05), with a 40 kb resolution. Hi-C gene annotation involved identifying interactions with gene promoters, defined as ±2 kb of a gene Transcription Start Site (TSS). This analysis identified 303,464 deoxyribonucleic acid (DNA)–DNA interactions used for our study.

### SNP to gene mapping for annotation and enrichment analyses

SNPs were mapped to genes using a combination of genome position information (i.e. closest gene), brain-specific expression Quantitative Trait Locus (eQTL) and Hi-C information.

Gene definitions were downloaded from Gencode (ftp://ftp.ebi.ac.uk/pub/databases/gencode/Gencode_human/release_32/GRCh37_mapping/gencode.v32lift37.basic.annotation.gtf.gz). Only genes with “protein_coding” biotype were included (20,076 unique gene symbols), to simplify interpretation of cellular mechanisms using pathway annotation information, which almost completely include only protein coding genes. Using chromatin state maps from the Roadmap Epigenomics project (Kundaje et al. 2015), we compiled a list of open chromatin and enhancer regions in brain tissue. These comprised maps derived from 13 human brain samples, including: neurospheres, angular gyrus, anterior caudate, germinal matrix, hippocampus, inferior temporal lobe, dorsolateral prefrontal cortex, substantia nigra, and fetal brain of both sexes (samples E053, E054, E067, E068, E069, E070, E071, E072, E073, E074, E081, E082, and E125), downloaded from http://www.roadmapepigenomics.org/. Open chromatin states were defined as genomic regions with epigenomic roadmap project’s core 15-state model values <=7. Enhancers were defined as those labeled with states “Enh” and “EnhG.”

For eQTL-based mapping, we searched for significant eQTLs in 12 types of brain tissue (GTEx v7: Amygdala, Anterior cingulate cortex BA24, Caudate basal ganglia, Cerebellar Hemisphere, Cerebellum, Cortex, Frontal Cortex BA9, Hippocampus, Hypothalamus, Nucleus accumbens basal ganglia, Putamen basal ganglia, and Substantia nigra) downloaded from https://www.gtexportal.org; Supplementary Note 1 (Battle et al. 2017). Of these, only SNPs overlapping open chromatin regions of brain-related samples (see previous paragraph) were included.

For 3D chromatin interaction mapping (Hi-C), we downloaded long-range chromatin interaction data from the adult cortex (Schmitt et al. 2016) and human developing brain (Won et al. 2016) (Interactions to TSS for cortical plate and germinal zone, Tables S22 and S23 of Won et al. (2016)). The enhancer region of these enhancer-promoter interactions was intersected with brain enhancers (see above) to only keep enhancer-promoter interactions overlapping known active brain enhancers. Then, the promoter region of these filtered enhancer-promoter interactions was mapped to a gene if it intersected with the region 250 bp upstream and 500 bp downstream of the corresponding gene transcription start site. SNPs were mapped to a gene if they overlapped the promoter of the filtered enhancer-promoter sites.

Finally, SNPs were positionally mapped to the nearest gene if the shortest distance to either transcription start site or end site was 60 kb. This cutoff was selected because it maps the majority (90%) of SNPs to their nearest gene, following a distance distribution analysis.

The order of SNP-gene mapping was as follows: SNPs that mapped to a gene via brain eQTL or Hi-C interactions were prioritized and not also positionally mapped to a gene. A SNP was allowed to map to genes using both eQTL and Hi-C. SNPs without eQTL or Hi-C mappings were positionally mapped to a gene. Where a SNP positionally mapped to multiple genes, all associations were retained. These SNP-gene mappings were used for the pathway and gene set enrichment analysis described below, as well as to annotate SNPs from the GWAS analysis.

Using these criteria, 7.7% of SNPs mapped to genes using nonpositional information (246,357 by eQTL and 16,923 by HiC, for a total of 263,280 SNPs); 2,917,948 SNPs mapped solely by positional information (89.2%). In total, SNPs mapped to 18,782 genes. 1,711,969 SNPs did not map to any genes (34.9%).

### Brain imaging analysis

Brain structure was assessed in a subset of the full sample that underwent magnetic resonance imaging on a 3 T Siemens TIM Trio scanner (1,000 individuals). T1-weighted structural magnetic resonance imaging (MRI) acquisitions were obtained with the magnetization-prepared rapid gradient-echo sequence with the following parameters: field of view =180 × 240mm; matrix = 192 × 256 × 160 slices; TR/TE/TI =1,810 ms/3.5 ms/1,100 ms; flip angle = 9; 1.0 mm slices. The diffusion weighted acquisitions used a twice-refocused spin-echo single-shot echo planar imaging (EPI) sequence with 64 diffusion weighted directions with *b* = 1,000 s/mm^2^, and seven scans with b = 0 s/mm^2^ in 2 mm slices (Satterthwaite et al. 2014). T1 weighted scans were processed with the CIVET processing pipeline (Version 1.1.12; Montreal Neurological Institute). To compute cortical thickness and surface area, CIVET performed linear registration to stereotaxic space and classification of tissue, and deformable surface models were used to create white and gray matter surfaces for each hemisphere with 40,962 vertices each (Lerch and Evans 2005). Diffusion weighted scan preprocessing involved correcting for motion, and eddy current distortions with FSLs eddy correct and calculating fractional anisotropy by fitting the diffusion tensor model in each voxel using FSL’s dtifit function. The TBSS pipeline was used to remove nonwhite matter (threshold of 0.2) and skeletonize each individual’s fractional anisotropy image (Smith et al. 2006). The association of significant SNP genotypes with average cortical thickness, cortical surface area, and white matter fractional anisotropy was assessed with linear models controlling for age, sex, and genetic ancestry using the first four principal components from SNP-based genotypes. Due to negligible numbers of individuals in the neuroimaging sample that were homozygous for minor alleles, minor allele carriers (homozygous or heterozygous) were compared to individuals who had two copies of the major allele. After excluding individuals not included in the genetic association analysis and those with poor quality scans (based on visual inspection), 191 (white matter)/267 (cortex) individuals for rs5765534 and 197 (white matter)/277 (cortex) for rs77601382 remained for inclusion in these analyses.

### Gene set enrichment analysis

For each of the nine CNB phenotypes, gene set enrichment analysis was performed using an implementation of Gene Set Enrichment Analysis (GSEA) for genetic variants (Wang et al. 2007; Wang et al. 2010). GSEA was selected as it computes pathway enrichment scores (ESs) using all available SNP information, which improves sensitivity, rather than using a hypergeometric model limited to SNPs passing a specific GWAS *P*-value cutoff. Moreover, pathway significance is ascertained using sample permutation, which corrects false-positives arising due to mapping of a few high-ranking SNPs to multiple nearby genes in the same pathway (Mooney et al. 2014). All SNPs were mapped to genes (as described in the “SNP-gene mapping for annotation and enrichment analyses” section above) and the gene score was defined as the best GWAS marginal *P*-value of all mapped SNPs for each gene. For each pathway, GSEA computes an ES using the rank-sum of gene scores. The set of genes that appear in the ranked list before the rank-sum reaches its maximum deviation from zero, is called the “leading edge subset”, and is interpreted as the core set of genes responsible for the pathway’s enrichment signal. Following computation of the ES, we created a null distribution for each pathway by repeating genome-wide association tests with randomly label-permuted data and by computing ES from these permuted data; in this work, we use 100 permutations to reduce computational burden. As a test of sensitivity to this parameter, we increased this value to 1,000 for the working memory phenotype (lnb_tp2). Finally, the ES on the original data is normalized to the score computed for the same gene set for label-permuted data (Z-score of real ES relative to mean of ES in label-permuted data), resulting in a Normalized Enrichment Score (NES) per pathway. The nominal *P*-value for the NES score is computed based on the null distribution and FDR correction is used to generate a q-value.

We used enrichment analysis to perform pathway analysis using pathway information compiled from HumanCyc (Romero et al. 2005) (http://humancyc.org), NetPath (http://www.netpath.org) (Kandasamy et al. 2010), Reactome (http://www.reactome.org) (Fabregat et al. 2016), NCI Curated Pathways (Schaefer et al. 2009), mSigDB (Subramanian et al. 2005) (http://software.broadinstitute.org/gsea/msigdb/), and Panther (Mi et al. 2005) (http://pantherdb.org/) and Gene Ontology (The Gene Ontology Consortium 2019) (Human_GOBP_AllPathways_no_GO_iea_May_01_2018_symbol.gmt, downloaded from http://download.baderlab.org/EM_Genesets/May_01_2018/Human/symbol/Human_GOBP_AllPathways_no_GO_iea_May_01_2018_symbol.gmt); only pathways with 20–500 genes were used.

We also used enrichment analysis to perform a brain system and disease analysis using brain-related gene sets we compiled from various literature sources (see Supplementary Table 5 and Supplementary Note 5). Brain system gene sets included those identified through transcriptomic or proteomic assays in human brain tissue (i.e. direct measurement of expression), and genes associated with brain function by indirect inference (e.g. genetic association of nervous system disorders); both groups of gene sets were combined for this enrichment analysis. The transcriptomic/proteomic gene sets included: genes identified as markers for adult and fetal brain cell types using single-cell transcriptomic experiments (Darmanis et al. 2015; Lake et al. 2016; Nowakowski et al. 2017), genes enriched for brain-specific expression [Human Protein Atlas project, https://www.proteinatlas.org (Yu et al. 2015)]; genes co-expressed with markers of various stages of human brain development [BrainSpan (Kang et al. 2011)]; and genes encoding proteins altered in the schizophrenia synaptosomal proteome (Velasquez et al. 2017). Brain disease gene sets included: genes associated with schizophrenia, bipolar disorder, autism spectrum disorder and major depressive disorder through large-scale genetic association studies by the Psychiatric Genomics Consortium (Schizophrenia Working Group of the Psychiatric Genomics Consortium 2014) (Supplementary Note 5); genes associated with nervous system disorders by the Human Phenotype Ontology (Kohler et al. 2019). Genes in the second group were filtered to only include genes with detectable expression in the fetal (Zhong et al. 2018) or adult human brain (Yu et al. 2015). A total of 1,321 gene sets were collected across both system and disease categories (Table S14). Only gene sets with 20–500 genes were included in the analysis; 421 gene sets met these criteria and were included in the enrichment analysis.

### Leading edge gene interaction network

Genes contributing to pathway enrichment results (leading edge genes) were obtained in our GSEA analysis for genetic variants (Wang et al. 2007). A gene-gene interaction network was constructed from leading edge genes of pathways with *q* < 0.05 using the online GeneMANIA service [v 3.6.0; https://genemania.org (Franz et al. 2018)] (human database, default settings); the resulting network and edge attributes were downloaded. This network was imported into Cytoscape v3.7.1 for visualization. Known drug associations were obtained from DGIdb (Cotto et al. 2018) and GWAS associations with nervous system disorders were obtained from the NHGRI-EBI GWAS catalog, via programmatic search using the TargetValidation.org API (Buniello et al. 2019; Carvalho-Silva et al. 2019). Cell type marker information was compiled from single cell RNA-seq datasets, including those for adult and fetal human brain (Darmanis et al. 2015; Lake et al. 2016; Nowakowski et al. 2017).

## Results

**Fig. 1:**
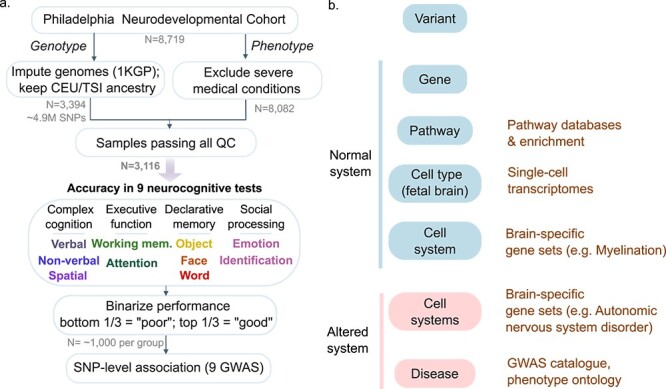
Framework for multi-scale systems genomics analysis for neurocognitive phenotypes from the PNC. (a) Workflow for GWAS. Genotypes were imputed (1,000 Genomes Project (1KGP) reference) and limited to European samples. Samples with severe medical conditions were removed and invalid test scores excluded. Nine neurocognitive test scores were binarized after regressing out age and sex. GWAS was performed using the accuracy measure as a phenotype for each of these nine phenotypes. (b) Framework to organize variant-level associations into a multi-scale systems view in health (blue) and disease (red). Existing functional genomic resources used for annotation shown in brown.

We developed a systems genomics analysis workflow to identify genetic variants associated with normative cognitive phenotypes (Fig. 1). Briefly, genotypes were imputed using a reference panel from the 1,000 Genomes Project (1000 Genomes Project Consortium et al. 2015), and samples were limited to those of European genetic ancestry (Supplementary Figs. 1–3, Supplementary Table 1). In total, 3,394 individuals and ~4.9 M SNPs passed the quality control and imputation process. Following quality control of phenotype data, 3,116 European samples passed both genotype and phenotype filters and were included in downstream analyses. We selected nine phenotypes from the Penn CNB representing overall accuracy in four cognitive domains: complex cognition, executive function, declarative memory, and social processing (Supplementary Table 3). Measures included performance for verbal reasoning, nonverbal reasoning, spatial reasoning, attention allocation, working memory, recall tests for faces, words and objects, and emotion identification (Gur et al. 2010). As age and sex is known to affect CNB task accuracy in 8–21 year olds, these variables were regressed out of the phenotype (Supplementary Table 4) and samples were thereafter binarized into poor and good performers (bottom and top 33% percentile, respectively) resulting in ~1,000 samples per group for each phenotype (Supplementary Figs. 4 and 5, Supplementary Table 3).

**Fig. 2:**
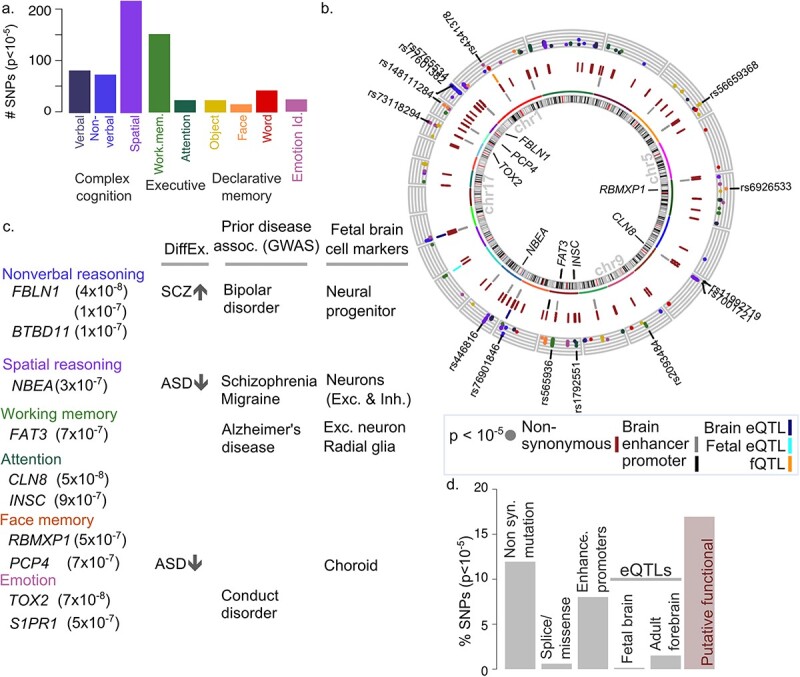
GWAS for neurocognitive phenotypes from the PNC. (a) Breakdown of SNPs achieving suggestive significance (*P* < 10^−5^), by phenotype (top). (b) Suggestive and significant SNPs and associated genes. The outermost ring shows the location of suggestive peaks (*P* < 10^−5^), colored by phenotype (see b); y-axis shows –log10 (SNP p), so that SNPs with stronger significance are higher. SNPs with *P* < 10^−7^ are labeled. The tracks with ticks indicate functional consequences of associated SNPs. The track closest to the middle indicate SNPs overlapping brain enhancers (light gray) or promoters (black). The dark red middle track indicates SNPs with nonsynonymous variation, including NMD transcript, missense or splice variants (BioMart) (Smedley et al. 2015). The outermost track indicates QTL associations, including eQTL in adult prefrontal cortex (dark blue), fetal brain (cyan), or neuronal cell proportions in the adult brain (fQTL; orange) [GTEx (Battle et al. 2017)]. Genes associated with top SNPs are indicated within the circle. See Supplementary Note 1 for annotation sources. (c) Genes associated with top SNPs (*P* < 3 × 10^−7^) with prior knowledge about relevance to brain development and psychiatric disorders. Columns indicate differential expression in neurodevelopmental disorders (Wang et al. 2018) (SCZ = schizophrenia; ASD = autism), significant association with a nervous system disorder (Buniello et al. 2019), or status as marker gene for specific cell types in fetal brain (Nowakowski et al. 2017). (d) Breakdown of functional consequence of top SNPs and by functional consequence (bottom). Consequence shown is limited to effect on protein sequence (Smedley et al. 2015), presence in enhancers or promoters in adult cortical regions (Kundaje et al. 2015), eQTL in fetal brain, or adult forebrain. Final bar shows cumulative proportion of putatively functional SNPs.

For each of the nine phenotypes, we first performed SNP-level GWAS, as a comparative baseline following traditional methods. We used a mixed linear models association analysis (MLMA) that included genome-wide genetic ancestry as a covariate [GCTA (Yang et al. 2011)]. Among the nine phenotypes, 661 SNPs had suggestive levels of significance at the genome-wide level (*P* < 10^−5^; Fig. 1b and c, Fig. 2a and b, Supplementary Figs. 6 and 7, Supplementary Table 6). Over half of these SNPs are associated with tasks related to complex cognition, i.e. verbal reasoning, nonverbal reasoning and spatial reasoning (377 SNPs or 57%). 27% were associated with executive function (177 SNPs), which included attention allocation and working memory. In total, 13% SNPs were associated with declarative memory tasks (83 SNPs), which included face recall, word recall, and object recall. A total of 4% of SNPs were associated with emotion identification (24 SNPs), a measure of social processing. More generally, SNPs associated with PNC cognitive phenotypes at suggestive significance levels (*P* < 10^−5^) map to genes previously associated with diseases of the nervous system and/or mark cell types in the fetal and newborn brain (Darmanis et al. 2015; Nowakowski et al. 2017) (Fig. 2c, Supplementary Table 7). We predict that one-sixth of suggestive peaks (112 SNPs or 17%) are linked to a functional consequence in brain tissue, including nonsynonymous changes to protein sequence (Fig. 2d), presence in brain-specific promoters and enhancers, or association with changes in gene expression (Supplementary Table 6).

**Fig. 3:**
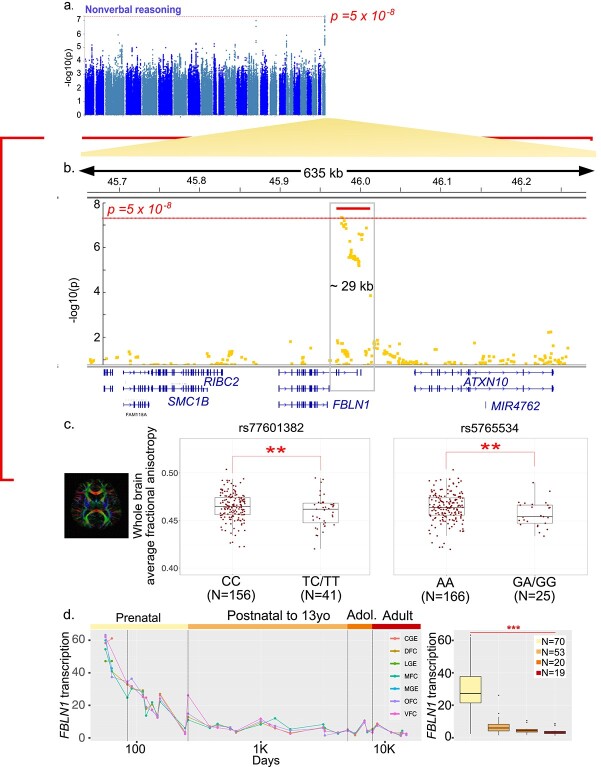
Genome-wide significance of *FBLN1* region for binarized performance in nonverbal reasoning (a) Manhattan plot of univariate SNP association with binarized performance in nonverbal reasoning (*n* = 1024 poor vs. 1023 good performers; 4,893,197 SNPs). Plot generated using FUMA (Watanabe et al. 2017). (b) Detailed view of hit region at chr22q13. Two SNPs pass genome-wide significance threshold, rs77601382 and rs74825248 (*P* = 4.64e – 8). View using integrated genome viewer [v2.3.93 (Robinson et al. 2011; Thorvaldsdottir et al. 2013)]. The red bar indicates the region with increased SNP-level association. (c) Association of significant *FBLN1* SNP genotypes with whole brain average white matter integrity (linear model, *P* < 0.025 for genotype term, after controlling for age, sex, and genetic ancestry; horizontal jitter added to points for visibility). (d) *FBLN1* transcription in the human brain through the lifespan; data from (Li et al. 2018). FPKM values are shown for central and medial ganglionic eminences (CGE, MGE), and dorsal, orbital, ventral, and medial frontal cortices (DFC, OFC, VFC, MFC). Right: FPKM values grouped into prenatal (<37PCW), postnatal (birth to <13YO), adolescent (13YO to <21YO), and adult periods (>21YO) (*P* < 2e – 16, one-way Analysis of Variance (ANOVA)).

Nonverbal reasoning was the only phenotype with SNPs passing the cutoff for genome-wide significance (rs77601382 and rs5765534, *P* = 4.6 × 10^−8^) (Fig. 3). The peak is located in a ~33 kb region (chr22:45,977,415-46,008,175) overlapping the 3′ end of the Fibulin-1 (*FBLN1*) gene, including the last intron and exon (Fig. 3b). The proportion of good and poor performers genotyped on each array platform was comparable (*P* > 0.1, chi-squared test). Poor performers have an increased proportion of the major C allele for rs77601382 (C/T) and the major A allele for rs5765534 (A/G). To better understand the significance of this gene in brain development, we examined the association between structural integrity of the brain’s white matter estimated from diffusion weighted MRI and these two SNPs. As expected, fractional anisotropy exhibited a robust positive relationship with age, reflecting the ongoing maturation of white matter throughout childhood and adolescence (Supplementary Fig. 8, *P* < 1 × 10^−8^ for both SNPs). For both SNPs, minor allele carriers had lower fractional anisotropy indicating less mature white matter integrity, compared with individuals with two copies of the major allele (multiple linear regression, *P* < 0.025, Cohen’s d > 0.43 for each SNP, Fig. 3c). There were no differences in cortical thickness or surface area between the two genotype groups (*P* > 0.25).

To better characterize *FBLN1*, we examined its expression in published bulk-tissue and single-cell human brain transcriptomes. Across the human lifespan, *FBLN1* transcription demonstrates highest relative expression in the early stages of fetal brain development (Fig. 3d; *P* < 2e – 16, one-way ANOVA), with little to no expression in the adult brain (Battle et al. 2017; Li et al. 2018) (Fig. 3d, Supplementary Fig. 9). Consistently, multiple single-cell transcriptomic studies of the prenatal human cortex report *FBLN1* to be a marker of dividing, intermediate progenitor cells (IPC) (Nowakowski et al. 2017; Bhaduri et al. 2021). This includes IPCs of the radial glial lineage resulting in newborn excitatory neurons, as well as those arising from the median ganglionic eminence (Nowakowski et al. 2017; Bhaduri et al. 2021). Additionally, *FBLN1* has been reported to be expressed in dividing cells, pericytes, outer radial glia, Cajal–Retzius cells, and microglia of the prenatal cortex (Polioudakis et al. 2019; Bhaduri et al. 2021). Consistent with this developmental pattern of expression, *FBLN1* is not reported to be a marker of cells in the adult human brain (Darmanis et al. 2015; Lake et al. 2016) (Fig. 3d, Supplementary Fig. 9) (Battle et al. 2017). *FBLN1* encodes a glycoprotein present in the extracellular matrix; this protein is a direct interactor of proteins involved in neuronal diseases, such as Amyloid Precursor Protein-1 (Ohsawa et al. 2001) [Supplementary Fig. 10 (Stark et al. 2006)]. *FBLN1* is overexpressed in *post-mortem* brain tissue of individuals with schizophrenia, but not in those diagnosed with bipolar disorder and autism spectrum disorder (Gandal et al. 2018), and has also been previously associated with genetic risk for hyperthymic temperament in bipolar disorder (Fig. 1d, (Greenwood et al. 2012; Wang et al. 2018)). We conclude that *FBLN1*, which contains genetic variants associated with nonverbal reasoning test performance, shows characteristics of a gene involved in neurodevelopment, the dysregulation of which could increase risk for neurodevelopmental and neuropsychiatric disorders (Brainstorm et al. 2018).

**Fig. 4:**
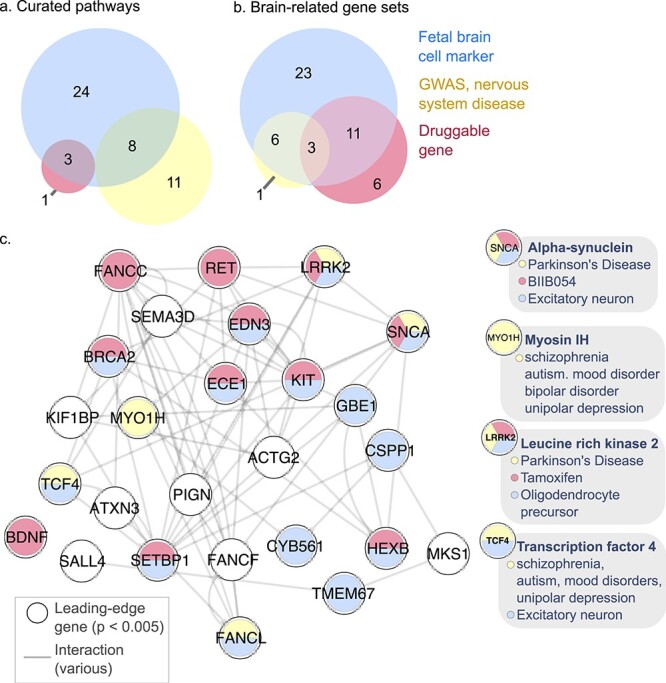
Pathway and brain system and disease analysis for the working memory performance phenotype (a) attributes of leading edge genes in pathway gene sets associated (*q* < 0.05) with working memory. Colors indicate transcription in brain cell types (blue), genetic associations with nervous system disorders (yellow), or those with known drug targets (pink) (*n* = 53 genes in total; 47 with annotations). (b) Leading edge genes in brain-related gene sets associated with disease, drugs or brain cell types (*n* = 71 genes total; 50 with annotations). Details in Supplementary Table 13. Legend as in (a). (c) Gene–gene interaction network for working memory leading edge genes from enriched (*q* < 0.05) brain-related gene sets. Only genes with top SNP *P* < 5 × 10^−3^ are shown (26 genes). Nodes show genes and fill color indicates genes associated with brain cell types, drugs or genetic associations with nervous system disorders (colors as in panel a, white indicates absence of association). Edges indicate known interactions [from GeneMANIA (Franz et al. 2018)]. Genes from the network with disease associations are highlighted with gray description bubbles.

Pathway analysis is an established systems genomics technique used to improve the statistical power of subthreshold univariate signal by aggregation of signal and reduction of multiple hypothesis testing burden, as well as to provide mechanistic insight into cellular processes that affect phenotypic outcome. Pathway analysis has been successfully used to link genetic disease risk to cellular processes for diseases in various domains, including schizophrenia (Schizophrenia Working Group of the Psychiatric Genomics Consortium 2014), breast cancer (Michailidou et al. 2017) and type 2 diabetes (Xue et al. 2018). We performed pathway analysis for the nine phenotypes using a rank-based pathway analysis strategy [GSEA (Subramanian et al. 2005; Wang et al. 2007), 500 permutations; 4,102 pathways tested]. SNPs were mapped to genes using brain-specific eQTL, chromatin interaction, and positional information, using the same method as described above. The working memory phenotype demonstrated significant enrichment of top-ranking genetic variants in a developmental pathway (*q* < 0.05; Supplementary Tables 8–10), showing biologically relevant signal where our univariate SNP-based baseline analysis did not. An advantage of the rank-based pathway analysis over those based on hypergeometric or binomial tests, is that the former provides a list of “leading-edge” genes driving the pathway-level enrichment signal, which can be further interpreted. We annotated leading edge genes with prior knowledge about genetic associations with nervous system disorders, transcription in brain cell types (Darmanis et al. 2015; Lake et al. 2016; Nowakowski et al. 2017; Buniello et al. 2019) and known drug interactions (Cotto et al. 2018). Out of 53 leading edge genes of this gene set, roughly one-half are known brain cell markers (25 genes or 47%), roughly one-third have known drug interactions (17 genes or 36%), and ~11% are associated with nervous system disease (six genes) (pathway *q* < 0.05, Fig. 4a, Supplementary Table 10, Supplementary Fig. 11). Among disease-associated genes were those associated with autism (*CSDE1*) and Parkinson’s disease (*LHFPL2*).

To perform a brain system and disease analysis, we performed a second enrichment analysis using gene sets curated from the literature, including transcriptomic and proteomic profiles of the developing and adult healthy brain and brains affected by mental illness, brain-related genome-wide association studies, and terms from a phenotype ontology (421 gene sets tested, Supplementary Note 5, Supplementary Table 5, Supplementary Data 1). Two gene sets pertaining to general nervous system dysfunction were significantly enriched (*q* < 0.05; GSEA, 500 permutations), again related to working memory (Fig. 4c, Supplementary Table 11). Roughly 17% of the 71 leading edge genes of these gene sets are associated with nervous system disorders (12 genes), roughly one-third have predicted drug targets (22 genes, 31%) and over half (43 genes or 61%) are markers of brain cell types (Fig. 4b and c; Supplementary Tables 12 and 13). Two genes have all three attributes: *SNCA* and *LRRK2* (Fig. 4c, Supplementary Table 13). Leading edge genes have genetic associations, including those with schizophrenia, autism spectrum disorder, Parkinson’s disease, Alzheimer’s disease, depression, and mood disorders (Fig. 4c, Supplementary Table 13). In summary, we identified many genetic variants associated with normative variation in neurocognitive phenotypes, enriched in pathways and gene sets related to development, nervous system dysfunction and mental disorders.

## Discussion

**Fig. 5:**
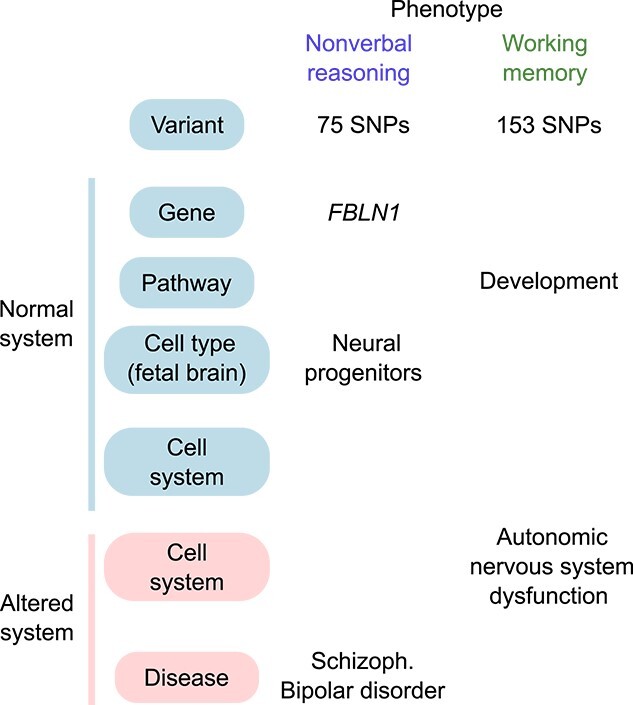
Summary of evidence linking genetic variants associated with cognitive test performance to multiple levels of brain organization. Each column shows data for an individual phenotype, grouped by phenotype domain; rows show associations at increasingly more general scales (from top to bottom); evidence linking variants to healthy system and disease system shown in blue and red, respectively. Pathways and cell systems are those identified by gene set enrichment analyses (*q* < 0.05). Cell types are those for which *FBLN1* is found to be a marker from single-cell transcriptome data (Nowakowski et al. 2017). Gene-disease associations are identified for significant SNPs, using gene-disease mappings from the NHGRI-EBI catalog (Buniello et al. 2019).

To our knowledge, this is the first study to identify genetic variants that may contribute to normal human variation in multiple, diverse cognitive domains, and to link these to various levels of brain system organization, including genes, pathways, cell types, brain regions, diseases, and known drug targets (Fig. 5). These associations, particularly potential drug targets, represent hypotheses to be experimentally validated in model systems to improve the mechanistic understanding of the molecular substrates of the respective phenotypes. While the UK Biobank (UKB) provides a valuable resource of genetics and cognitive assessments for a much larger sample size of 500,000 individuals, the participant age is 40–70 years, capturing brain changes in older adults. It is worth noting that the Penn CNB finds positive correlation of test accuracy with age in 8–21 year olds, and negative correlation with an initial sample of 18–84 year olds, which likely shows the relative effect of brain development versus aging on cognition (Gur et al. 2012). Unlike the Penn CNB, the UKB cognitive assessment test battery was not designed using established metrics such as test–retest validity and construct validity, which were measured after test administration (Fawns-Ritchie and Deary 2020). Test scores from the UKB cognitive assessment show variable correlation with scores from standardized assessments that intend to measure the same psychological construct (Fawns-Ritchie and Deary 2020).

Swagerman et al. measured the common variance from all tasks in the Penn CNB and compared this common factor to a Weschler Intelligence Scale for Adults-based general factor of intelligence (g-WAIS) (Swagerman et al. 2016). Using an oblique two-factor model of overlap in variance, they found that the common CNB factor completely overlapped the common g-WAIS factor with a correlation of 1.0. This overlap suggests that overall performance on the CNB correlates well with general intelligence (or “g”) as measured by a psychometric intelligence test battery (Ref 1). Consistent with this observation, the *FBLN1* locus was not significantly enriched in a large GWAS study of general cognitive ability (Savage et al. 2018).

We found an enrichment of genetic variants associated with complex cognitive phenotypes (75–219 suggestive peaks in a Manhattan plot), consistent with heritability estimates of up to 0.30–0.41 for these phenotypes (Robinson et al. 2015). We also found that many variants, genes, and pathways associated with normal variation in neurocognitive phenotypes have known roles in neurodevelopment, modulating gene expression in the fetal and adult brain and increasing risk for psychiatric diseases of neurodevelopmental origin (Fig. 1, Supplementary Tables 6, 7, 10, and  13). We found a significant association of *FBLN1* for nonverbal reasoning ability, as measured by the Penn Matrix Analysis Test (PMAT). Multiple lines of evidence suggest that *FBLN1*, the gene we find associated with genome-wide significant SNPs for nonverbal reasoning, is dysregulated in brain-related disease. In addition to the evidence provided in our results (Fig. 2 and Fig. 3c, Supplementary Figs. 9 and 10), the *FBLN1* gene has been associated with a rare genetic syndrome that includes multiple cognitive impairments, and protein levels of FBLN1 have been associated with altered risk for ischaemic stroke (Palumbo et al. 2018; Vadgama et al. 2019). However, the mechanism by which *FBLN1* contributes to normal brain function is not known. We also do not exclude the possibility that the genetic polymorphisms we identified within *FBLN1* may affect the function of neighboring or otherwise linked genes, which may instead or in combination affect the phenotype.

A limitation of the current study is the relatively small size of the patient cohort—roughly 1,000 cases and controls each per phenotype—compared with contemporary GWAS studies which may include over 100,000 individuals. The reduced sample size is partly because we chose to limit the analysis to individuals with European genetic ancestry, to maintain the largest number of samples while avoiding the confound with genetic ancestry. Furthermore, we dichotomized the phenotype into bottom and top performers, ignoring samples in the middle, as our goal was to work with a subset enriched for extremes within typical phenotypic variation, to strengthen signal. For all phenotypes tested in this work, we also performed genome-wide association tests using continuously valued measures, instead of binarized phenotypes; none of the associations resulted in significant results (data not shown).

This work contributes toward an understanding of the molecular and systems-level underpinnings of individual cognitive tasks that have been associated with specific brain systems. These associations will need to be validated in better-powered datasets, possibly using newer neurobehavioral measurement standards in the field (Weintraub et al. 2013) but can currently be used as hypotheses to plan biological experiments, or as support for orthogonal methods studying the relevance of genes and pathways we identify for brain biology. Studying the overlap in genetic architecture between these phenotypes, similar to cross-disorder genetic studies (Lee et al. 2013), may also inform disease classification (Jeste and Geschwind 2014; Clementz et al. 2016). Our analysis is limited to univariate genetic effects, and future work should explore the contribution of interactions between individual SNPs (Wang et al. 2017), though this will require many more samples per phenotype. We propose that research frameworks for linking genotype to phenotype for brain-related traits include systems genomics analysis, considering pathways, cells, anatomical structures, and physiological processes as organizational layers to improve the amount of genetic signal that can be extracted from available genetic data, which otherwise would be missed if just considering SNPs and genes. For example, the working memory phenotype had no significant SNPs that met the genome-wide significance cutoff. However, gene sets related to development and autonomic nervous system dysfunction demonstrated significant clustering of high-ranking variants, including those in *SLIT3* (rs62376937) and *ROBO2* (rs12497629), which mediate axon guidance in the developing nervous system. The conceptual strategy we outline in this work, of organizing variant-related annotation into a systems-level view is generalizable across biomedical domains and to human disease (Fig. 1 and Fig. 5). Integration of such evidence across studies can identify common themes or discrepancies to encourage thinking of a systems-level view of genotype–phenotype association for disease.

Genetic data used in this study were downloaded from dbGaP (phs000607.v1). Software written to perform the analysis in this manuscript have been made available under the MIT license at https://github.com/BaderLab/PNC_GeneticsofCognition. GWAS summary statistics for binarized phenotypes tested have been deposited at Zenodo under DOI:10.5281/zenodo.7843900.

## Supplementary Material

FINAL_PNC_Supp_bhad142

FINAL_PNC_SuppTables_bhad142

## CRediT authors statement

Shraddha Pai (Conceptualization, Data curation, Formal analysis, Methodology, Writing-original draft, Writing-review & editing), Shirley Hui (Formal analysis, Methodology), Philipp Weber (Formal analysis), Soumil Narayan (Formal analysis), Owen Whitley (Formal analysis), Peipei Li (Formal analysis, Resources), Viviane Labrie (Resources), Jan Baumbach (Supervision), Anne Wheeler (Formal analysis, Methodology), Gary Bader (Conceptualization, Methodology, Project administration, Supervision, Writing-original draft, Writing-review & editing).
