# Correction to: Multi-scale systems genomics analysis predicts pathways, cell types, and drug targets involved in normative variation in peri-adolescent human cognition

**DOI:** 10.1093/cercor/bhad223

**Published:** 2023-06-08

This is a correction to: Shraddha Pai and others, Multi-scale systems genomics analysis predicts pathways, cell types, and drug targets involved in normative variation in peri-adolescent human cognition, *Cerebral Cortex*, 2023, bhad142, https://doi.org/10.1093/cercor/bhad142

In the originally published version of this manuscript, there was an error in the caption to Figure 4. This should read: “Details in Supplementary Table 13” instead of: “Details in Supplementary Table.”

In the caption to Table 5, the third sentence was erroneous and was deleted: “Circles indicate relative number of suggestive variant peaks (p < 10-5) from GWAS)”.

These errors have been corrected in the article.

